# Rotary Friction Welding of Molybdenum without Upset Forging

**DOI:** 10.3390/ma13081957

**Published:** 2020-04-22

**Authors:** Miaoxia Xie, Xiangtao Shang, Yanxin Li, Zehui Zhang, Minghui Zhu, Jiangtao Xiong

**Affiliations:** 1 School of Mechanical and Electrical Engineering, Xi’an University of Architecture and Technology, Xi’an 710055, China; shang_x_t@163.com (X.S.); li_y_x@163.com (Y.L.); KKLebron23@163.com (Z.Z.); zhu_m_h@163.com (M.Z.); 2 School of Materials Science and Engineering, Northwestern Polytechnical University, Xi’an 710072, China; xiongjiangtao@nwpu.edu.cn

**Keywords:** molybdenum, rotary friction welding, microstructure, mechanical properties

## Abstract

A large instantaneous axial forging load is required to be applied for the final stage of rotary friction welding (RFW), which is usually conducive to obtaining clean, compact, and high-quality joints. However, for slender fuel claddings made of molybdenum (Mo) with low stiffness, the instantaneous axial forging load cannot be applied at the final stage of welding. This study carried out RFW tests without upset forging on Mo in the atmospheric environment and investigated the effects of welding time on joint morphology, axial shortening, microstructures, microhardness, tensile strength, and tensile fracture morphology. It found that the excessive and abrupt burning and a lot of smoke were generated around the weld zone during welding and spiral flashes were observed after welding. Under welding pressure of 80 MPa and spindle speed of 2000 r/min, the minimum average grain size and maximum tensile strength can be obtained in 4 s when the welding time is between 2–5 s. Scanning electron microscope (SEM) results show that there were morphologies of a large number of intergranular fractures and a small number of transgranular fractures in the fracture. The above results demonstrated that it is feasible to use RFW without upset forging to seal the last weld spot on upper end plugs of fuel claddings made of Mo in high-pressure inert gas, which would not only obtain reliable welding quality but also seal high-pressure inert gas in cladding tubes. The research results have a practical guiding significance of manufacturing accident-tolerant Mo nuclear fuel cladding.

## 1. Introduction

Zirconium alloy is widely used to produce nuclear fuel claddings and core structural parts [1,2,3]. However, when the temperature exceeds 1200 °C, zirconium can react with water vapor to produce large amounts of hydrogen, easily causing explosions, and release large amounts of heat, further accelerating the melting of a reactor core [4,5]. Molybdenum (Mo) has advantages, such as high melting point (2610 °C), small cross-section area of neutron absorption, small linear expansion coefficient, good high-temperature strength, high thermal conductivity, and excellent corrosion resistance. Therefore, Mo is used to prepare nuclear fuel claddings that can withstand serious accident conditions for a long time [6]. Replacing traditional nuclear fuel rod claddings with Mo claddings can improve the safety of nuclear power, which has been increasingly concerned with relevant researches in recent years [7,8,9]. Welding is a key step in preparing nuclear fuel cladding tubes. Due to the large size of the weld seam, heat-affected zone, and severe grain coarsening after welding Mo, the joints show poor strength and toughness under the combined actions of intrinsic brittleness of the materials and weakening effect of impurity segregation on grain boundaries [10,11]. In addition, the hollow upper end plugged hole of nuclear fuel claddings needs to be welded and sealed in a high-pressure helium atmosphere, so as to fill a certain pressure (2–3 MPa) in fuel rods to counteract with the in-service water pressure in the nuclear reactor [12,13].

In atmospheric pressure laser welding of molybdenum, high-quality joint performance can be obtained due to the high-energy density of the heat source, the small heating zone, and the narrow and deep weld seams [14,15,16,17]. However, when Mo is welded with laser under high pressure on 2–3 MPa, the weld penetration largely decreases, while the width of heating zone greatly increases, leading to the poor performance of joints or failure of welding [18,19]. Rotary friction welding (RFW) is a solid-state welding method [20,21], during which the materials do not melt and temperature is lower (with respect to laser welding), which is very beneficial to improve mechanical properties of Mo joints that are very sensitive to heat input. Furthermore, RFW process and welding quality are not affected by environmental pressure. Andrzej Ambroziak et al. [22] studied RFW of titanium–zirconium–molybdenum (TZM) alloy and dissimilar joints of metals, like vanadium, tantalum, niobium, titanium, and copper, welded through RFW. It is worth noting that in order to prevent reactions, like oxidation of Mo alloy during welding in atmospheric environment, all RFW tests were conducted in liquid (electric spark machining oils) by Andrzej Ambroziak et al. This undoubtedly plays a very good role in inhibiting excessive grain growth of the weld zone, which is worth learning from. Unfortunately, they did not do a detailed analysis of the mechanical and material properties of the joint. B. Tabernig et al. [23] did research in the field of RFW of molybdenum–hafnium–carbon (MHC) alloy and TZM alloy. It was found that the weldability of MHC is better than that of TZM alloy. M. Stutz et al. [24] conducted comparative research on inertia friction welding of pure Mo and TZM and found that it is more difficult to perform inertia friction welding of pure Mo. The reasons are shown as follows: On the one hand, thermal conductivity of Mo is very high; on the other hand, strength of pure Mo is lower than TZM at high temperature. Therefore, larger plastic deformation is easily produced during welding of pure Mo, resulting in poor welding quality and stability. By utilizing electron backscattered diffraction (EBSD) method, M. Stutz et al. [25] systematically compared and studied characteristics of microstructural evolution of friction welded joints of pure Mo and TZM.

**Figure 1:**
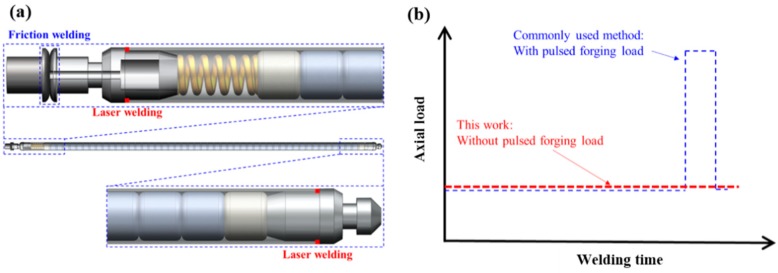
(**a**) Schematic diagram of welding nuclear fuel claddings made of molybdenum (Mo) and (**b**) characteristic of the axial load employed in this work.

In the above-mentioned studies on RFW or inertial friction welding of Mo, the researchers took forging measures at the final stage of welding. The length and outer diameter of nuclear fuel claddings were about 4 m and 8–16 mm, and wall thickness of the cladding tubes was not larger than 1 mm, so it was not suitable to apply forging load with impact when welding Mo claddings. Therefore, authors proposed a complete set of welding and encapsulation methods for Mo accident-tolerant nuclear fuel rods (Figure 1a). Specifically, the laser welding method was firstly used to realize the connection to girth welded between the upper end plugs and the cladding tubes as well as the lower end plugs and the cladding tubes under normal pressure/negative pressure. Finally, the upper end plugs with holes were welded and sealed using RFW without upset forging in a high-pressure helium atmosphere. Research on laser welding of Mo claddings can be found in research published elsewhere [8,9]. This study focused on the feasibility of using RFW without upset forging to seal the last weld spots on the upper end plugs of Mo fuel claddings, so as to provide guidance for developing reliable welding technology for fuel claddings made of Mo.

## 2. Test Materials and Methods

Pure Mo rods (Mo1) with diameter φ of 25 mm and axial length of 80–110 mm prepared through powder metallurgy and rolling were used in the tests and the contents of components are shown in Table 1. Before welding, the rods were cleaned with acetone. A C320A RFW machine (Hanzhong, China) was used with welding pressure of 80 MPa and spindle speed of 2000 r/min, which were almost the ultimate load and ultimate speed thereof. Considering high melting point and high-temperature strength of Mo, this study conducted RFW tests without applying forging load at the final stage of welding at different welding times for unchanged welding pressure of 80 MPa and spindle speed of 2000 r/min. The parameters of RFW process are listed in Table 2. In this way, at the final stage of welding, a small and constant load was utilized instead of a pulsed forging load (Figure 1b).

**Figure 2:**
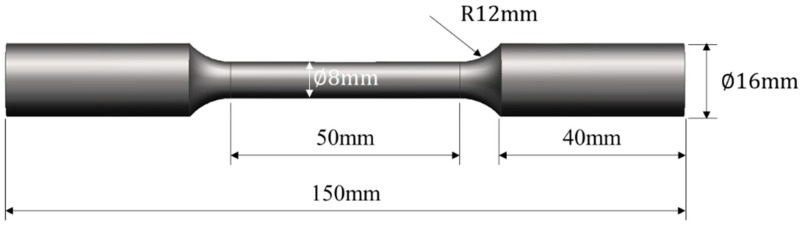
Schematic diagram of size of a tensile specimen.

After welding, cross-sectional metallographic specimens (the cross-section was parallel to the axis of the specimen) from the welded joints were prepared for standard procedures. Then, the specimens were etched with sodium hydroxide solution and potassium ferricyanide solution (with mass fractions both of 10%) according to the ratio of 1:1 for 60 s. By utilizing a Nikon EclipseMA200 optical microscope (Nikon, Tokyo, Japan) and a TESCAN VEGA II XMU scanning electron microscope (SEM, Brno, South Moravia, Czech), macro-morphologies and microstructures of cross-sections of the joints were observed. The tensile strengths of base metal and the welded joints were separately measured by a universal mechanical testing machine. The size of a tensile specimen is shown in the Figure 2 and tensile speed was 1 mm/min. A HXD-1000TMC/LCD Micro-Vickers hardness tester (Everone, Shanghai, China) was used to measure distribution of microhardness of cross-sections of the joints under the load of 300 gram force (gf), which was kept for 15 s. The micro-morphologies of tensile fractures of the joints were observed by employing the SEM.

## 3. Results and Discussion

### 3.1. Excessive and Abrupt Burning and Instability of Flashes during Welding

**Figure 3:**
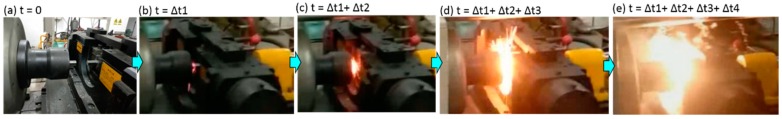
RFW experiment process of Mo: (**a**) time = 0; (**b**) time = △t1; (**c**) time = △t1 + △t2; (**d**) time = △t1 + △t2 + △t3; (**e**) time = △t1 + △t2 + △t3 + △t4.

**Figure 4:**
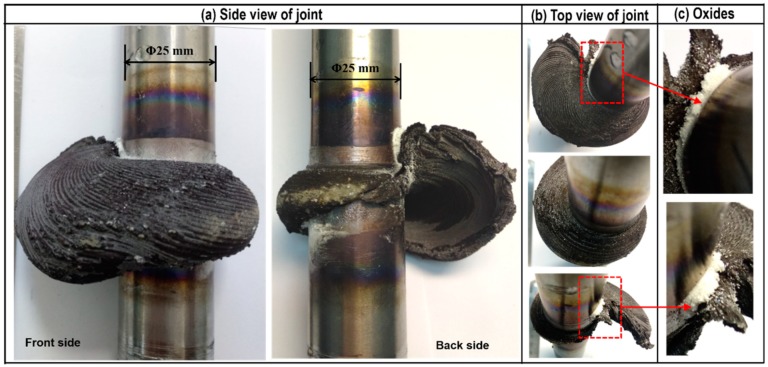
Instability of flashes on the Mo specimens obtained through RFW: (**a**) side view of joint; (**b**) top view of joint; (**c**) oxides.

Figure 3 shows the RFW experiment process of Mo at different times. It can be seen from the figure that excessive and abrupt burning-like phenomenon appeared around the weld zone during the RFW, accompanied with a lot of smoke generated. Figure 4 illustrates the typical joint morphologies obtained after welding. As shown in the figure, the flashes of the Mo-RFW joints were large and appeared in the asymmetrical, spiral shapes. Considering excessive motor torque during RFW of Mo, resulting in overloading motor in the test, the cause for formation of spiral flashes was probably that high melting point and thermal conductivity of Mo leading to unstable and plastic deformation instability during RFW. Moreover, due to the centrifugal action during high-speed rotation, the spiral flashes were formed. In addition, owing to RFW performed in air, a large number of white oxides powders were produced during welding, as shown in Figure 4c. In general, compared with RFW of steel, RFW of Mo has an obviously smaller process window and slight changes of welding parameters can exert great influences on joint morphology and axial shortening.

### 3.2. Effects of Welding Time on Macro-Morphology and Axial Shortening of the RFW-Mo Joints

**Figure 5:**
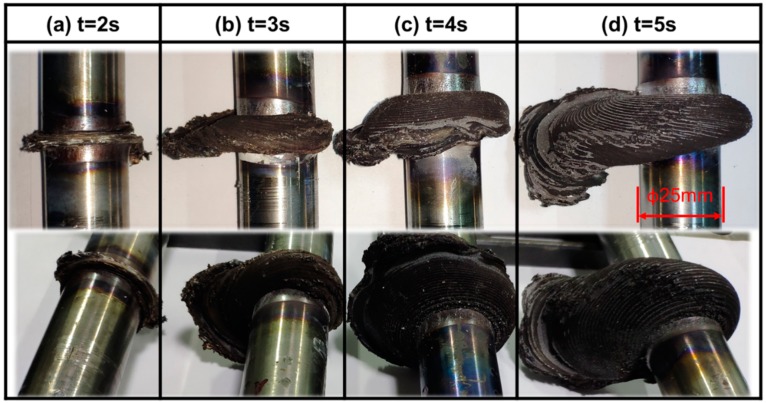
Macro-morphologies of the Mo joints welded through RFW at (**a**) t = 2 s; (**b**) t = 3 s; (**c**) t = 4 s; and (**d**) t = 5 s under unchanged welding pressure of 80 MPa and spindle speed of 2000 r/min.

Figure 5 demonstrates macro-morphologies of the welded joints obtained at 2, 3, 4, and 5 s under unchanged welding pressure of 80 MPa and spindle speed of 2000 r/min. It can be observed from the figure that with the increase of welding time, the volume of flashes gradually enlarged and spiral shape became more obvious.

**Figure 6:**
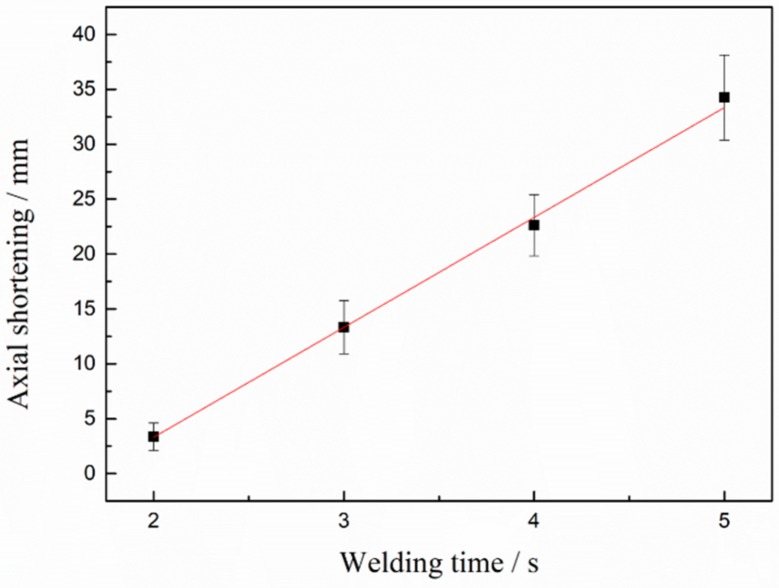
Effects of increase of welding time on axial shortening of the Mo joints welded through RFW under constant welding pressure of 80 MPa and spindle speed of 2000 r/min.

Figure 6 shows the influences of increase in welding time of axial shortening of the welded joints under unchanged welding pressure of 80 MPa and spindle speed of 2000 r/min. As demonstrated in the figure, with gradual increase of the RFW time of Mo from 2 s to 5 s, axial shortening monotonously rose. When welding time was 2 s, axial shortening was about 3 mm, and it reached about 33 mm at 5 s, which implies that RFW of Mo has a relatively narrow process window and slight changes of welding parameters can have great influences on joint morphology and axial shortening. It can be seen from the figure that axial shortening linearly rose with the increase of welding time, which can bring a lot of conveniences to accurately predict and control axial shortening in production. Under the conditions considered in this work, the formula for calculating axial shortening was y = 10.01x − 16.71. Here, y is axial shortening and x is welding time.

### 3.3. Influences of Welding Time on Structural Morphologies of Cross-Sections of the RFW-Mo Joints

**Figure 7:**
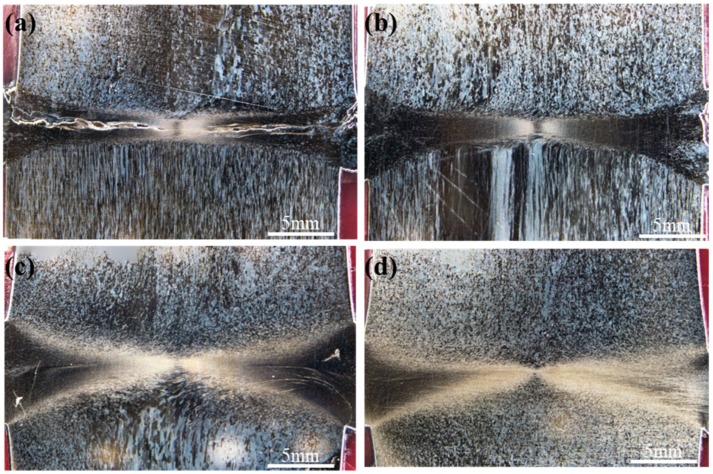
Joint morphologies of the RFW-Mo specimens under welding pressure of 80 MPa and spindle speed of 2000 r/min for (**a**) 2 s, (**b**) 3 s, (**c**) 4 s, and (**d**) 5 s.

**Figure 8:**
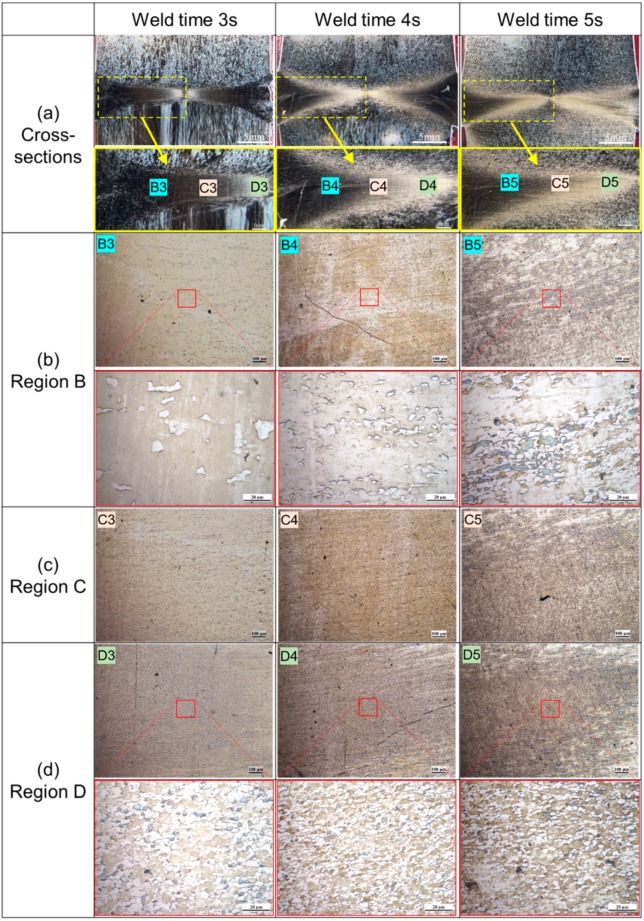
Metallographic microstructures of the Mo-RFW joint welded under welding pressure of 80 MPa and spindle speed of 2000 r/min for welding time 3 s to 5 s: (**a**) cross-sections; (**b**) region b of (**a**); (**c**) region c of (**a**); (**d**) region d of (**a**).

Figure 7 displays macro-morphologies of cross-sections (i.e., parallel to the axis of specimens) of the welded joints at 2, 3, 4, and 5 s under unchanged welding pressure of 80 MPa and spindle speed of 2000 r/min. The effects of welding time of bonding quality of interfaces were firstly discussed. As shown in Figure 5, obvious gaps near friction interfaces were observed on the cross-sections of the joints at welding time of 2 s (Figure 7a), while there were no gaps on the cross-sections of the welded joints obtained at 3, 4, and 5 s (Figure 7b–d). This indicates that welding time of 2 s is too short and the generated heat is not enough to realize metallurgical bonding with the workpieces. Figure 8 shows enlarged views of the areas near friction interfaces on the cross-sections of the joints after welding for 2 s. It can be seen that a large number of incomplete bonding defects existed on the interfaces after friction welding. Welding time affects frictional heat, and further influences plastic deformation and flow of the metal near the weld zone. Therefore, with the rise of welding time, expansion and deformation along the radial direction (i.e., horizontal direction in Figure 7) of the metal near the friction interfaces gradually occurred (Figure 7) and size of flashes (Figure 5) and axial shortening (Figure 6) monotonously rose.

Furthermore, by observing structural morphologies of the areas below friction interfaces on the cross-sections of the joints in Figure 7, morphology of fine as-rolled microstructures was found below the friction interfaces at 2 s, while a coarse one was shown at 3 s. When welding time was 4 s, a small number of residual morphologies of as-rolled microstructures with distortion and deformation were presented below the friction interfaces, and the morphologies of as-rolled microstructures could hardly be observed below friction interfaces at 5 s. When observing structural morphologies of the areas above friction interfaces on the cross-sections of the joints in Figure 7, it can be seen that there was no morphology of rolled microstructures above the friction interfaces of the four joints. The reason is that the workpiece on one side (i.e., below the friction interfaces in Figure 7) rotated at a high speed, while the other workpiece on the opposite side did not rotate during RFW of Mo. Owing to convective heat transfer coefficient of the rotary workpiece with surrounding air was much larger than that of the opposite workpiece, heat dissipation conditions of the workpieces on both sides of the friction interface were quite different, thus leading to difference of the workpieces in temperature field. The workpiece rotating around a high speed showed good heated dissipation conditions and low temperature, so as-rolled microstructures were more easily shown. Due to worse heat dissipation conditions and high temperature of the workpiece that did not rotate, recrystallization more easily occurred and as-rolled microstructures were more likely to change into equiaxial ones.

Microstructures near the friction interface exerted important influences on mechanical properties of the welded joints. Figure 8 shows microstructures of cross-sections of the welded joints at 3, 4, and 5 s under constant welding pressure of 80 MPa and spindle speed of 2000 r/min. In Figure 8, the horizontal coordinate represents the welding time and the vertical coordinate represents the different positions of the joint metallography. Because the average grain size gradually increased from the radial center to the edge, we omitted the enlarged pictures of Figure 8c Region C for the convenience of typesetting and display effect. As illustrated with the figures, in the dark areas near the friction interfaces, average grain size gradually increased along the radial direction from the center to surface of the specimens (Regions B, C, and D in Figure 8). Fine equiaxial microstructures were found in the dark areas near the center of the specimens (Region D in Figure 8). The average grain size in the Region D on cross-sections of the four Mo-RFW joints welded for 2, 3, 4, and 5 s were obtained according to standard methods of Determination of Estimating the Average Grain Size of Metal (ASTM E112-2013 Standard) [26].

**Figure 9:**
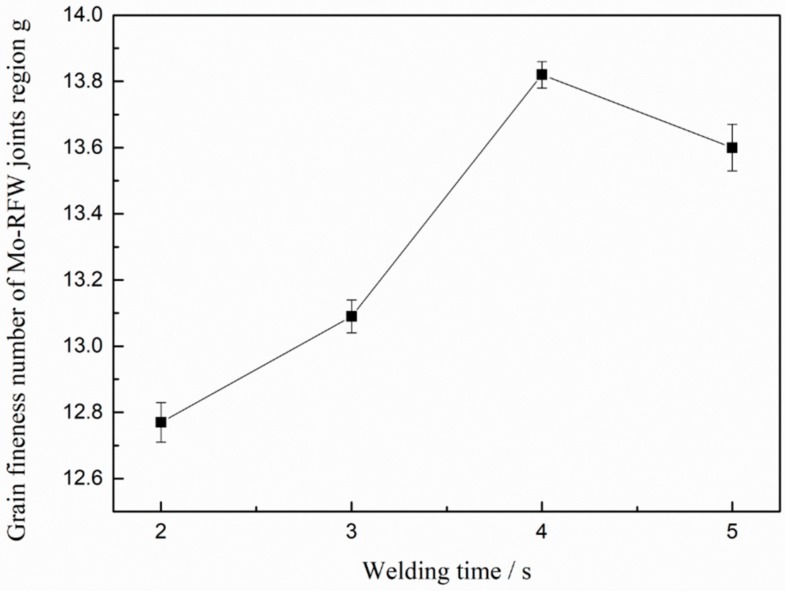
Effects of welding time on grain fineness number in central regions of friction interfaces of the Mo-RFW joints.

Figure 9 demonstrates influence of welding time on grain fineness number in Region D in Figure 8 on cross-section of the Mo-RFW joints welded according to the Determination of Estimating the Average Grain Size of Metal (ASTM E112-2013 Standard). It can be seen from the figure that with the gradual increase in RFW time of Mo from 2 s to 5 s, grain fineness numbers in the Region D on cross-sections firstly rose and then reduced. Grain fineness refers to the number of grains per unit area. Generally, the larger the grain fineness is, the more the number of grains per unit area is and the smaller the average grain size is. After being welded for 4 s, grain fineness number was maximum, that is, the average grain size was minimum. This means that too-long welding time will be harmful to the mechanical properties of the joint. Too-long welding time can not only increase the costs and reduce production efficiency, but also raise grain size, which may weaken mechanical properties of the joints.

### 3.4. Impacts of Welding Time on Microhardness of Cross-Sections of the Mo-RFW Joints

**Figure 10:**
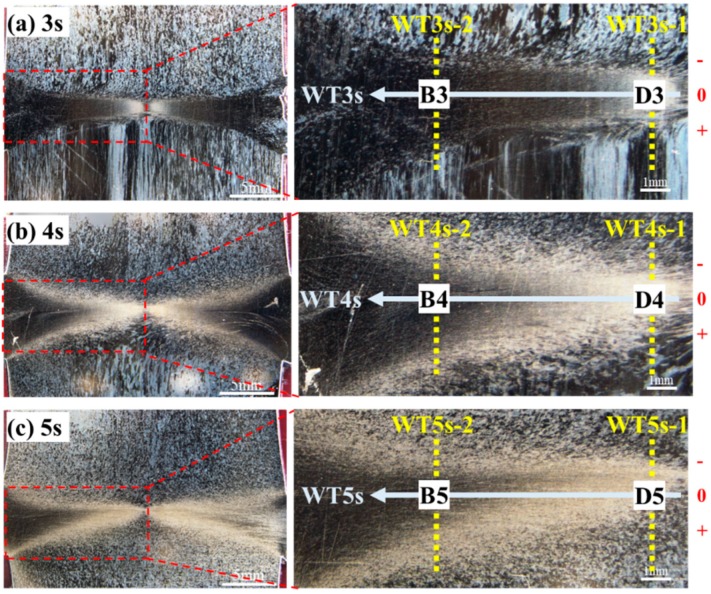
Schematic diagram of test scheme for microhardness of cross-sections of the Mo-RFW joints: (**a**) welding time 3 s; (**b**) welding time 4 s; (**c**) welding time 5 s.

The schematic diagram of the test scheme for microhardness of cross-sections of the Mo-RFW joints is shown in Figure 10 and three paths for marking microhardness were drawn on cross-sections of each joint. In the following discussions, WT3s-1, WT4s-1, and WT5s-1 refer to the test paths along axial direction at the position of Region D on cross-sections of the joints in Figure 8; WT3s-2, WT4s-2, and WT5s-2 indicate the test paths along axial direction at the position of Region B on cross-sections of the joints in Figure 8; and WT-3s, WT-4s, and WT-5s represent the test paths along with friction interfaces on the cross-sections of the joints.

**Figure 11:**
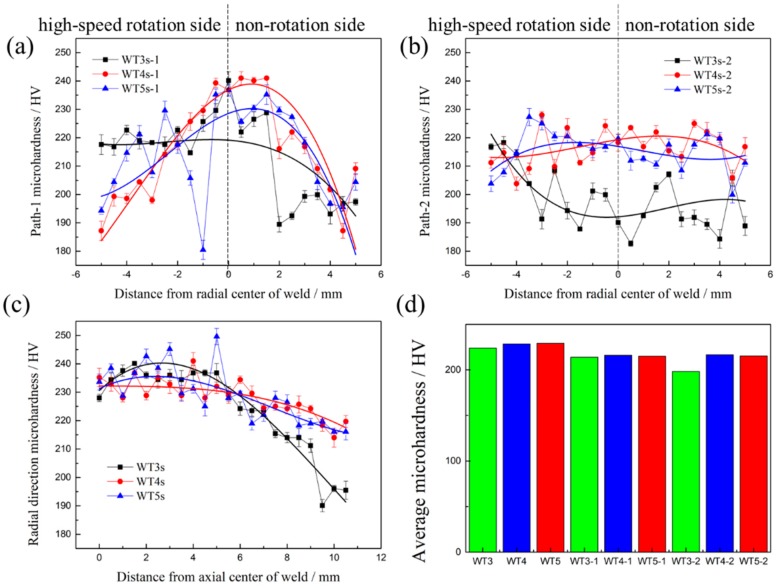
Microhardness distribution at different positions of the joints: (**a**) path-1; (**b**) path-2; (**c**) radial direction; (**d**) average microhardness.

Heating temperature is the most important factor affecting the microstructure evolution of materials in welding zone. S. Primig et al. studied the heating rate for the recrystallization behavior of molybdenum by continuous heating experiments of cold-compressed specimens with linear heating rates for the range of 1–1000 K/min to target temperatures between 800 and 1300 °C [27]. It was observed under both high- and low-heating rate that the volume fractions of recrystallized grains decreased with the increasing of target temperature, which led to that the higher the target temperature, the lower the microhardness. From the hardness test results along the “WT3s, WT4s, WT5s” path in the three joints with welding time of 3 s, 4 s, and 5 s in Figure 11c, it can be seen that the microhardness on the test path decreased with the increase of the distance to the central axis of the sample. This is mainly because the closer the workpiece was to the surface of the sample in the process of rotation, the greater the linear velocity, so the closer the workpiece was to the surface of the sample, the higher the friction heat and temperature. In addition, based on the test results from microhardness under the paths of WT3s-1 in Figure 11a and WT3s-2 in Figure 11b, it can be observed that microhardness at the left end of the curve was obviously higher than that at the right end. The reason is that the test area corresponding to the left end of the curve was located on the side of high-speed rotation during RFW, while the test area at the right end of the curve was on the nonrotation side during RFW. Compared with the nonrotation side, the high-speed rotation side had stronger convection heat transfer which resulted in lower temperature at high-speed rotation side. Figure 11d demonstrates the average microhardness under all paths. It can be observed from Figure 11d that microhardness under paths of WT3s-1 and WT3s-2 was obviously different, indicating that microhardness changed obviously along radial direction when welding for 3 s. Microhardness under paths of WT4s-1, WT4s-2, WT5s-1, and WT5s-2 showed no obvious difference, suggesting that microhardness along the radial direction changed slightly after welding time reached 4 s. Furthermore, it is evident that values obtained under the paths of WT3s, WT4s, and WT5s were slightly larger than those of the other six paths, which means that microhardness near the friction interfaces was larger than that on both sides of the interfaces.

### 3.5. Effects of Welding Time on Mechanical Properties of the Mo-RFW Joints 

**Figure 12:**
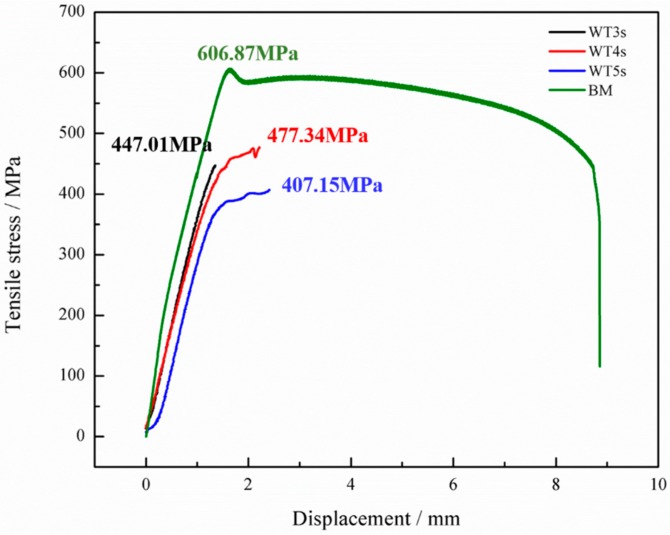
Tensile strength of the Mo-RFW specimens.

Figure 12 demonstrates tensile test results of the Mo-RFW joints. It can be seen from the figure that tensile strength of Mo base metal was 606.87 MPa. When welding time was 3 s, tensile strength of the joint was 447.01 MPa, which reached 73.65% that of the base metal. After welding for 4 s, tensile strength of the joint rose to 477.34 MPa, which was 78.66% that of the base metal. Moreover, as welding time was 5 s, tensile strength of the joint decreased to 407.15 MPa, reaching 67.09% that of the base metal.

**Figure 13:**
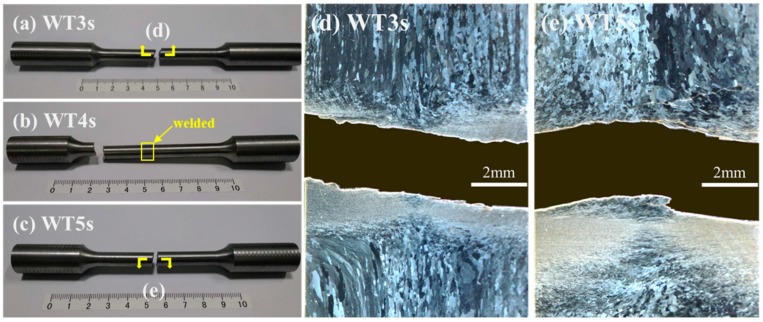
Positions of tensile fractures of the RFW-Mo joints: (**a**) welding time 3 s; (**b**) welding time 4 s; (**c**) welding time 5 s; (**d**) welding time 3 s partial enlarged view; (**e**) welding time 5 s partial enlarged view.

The joints welded for 3 s and 5 s were fractured at weld seams (Figure 13a,c), while the joint welded for 4 s was fractured in a parallel section far away from the weld seam (Figure 13b). With the increase in welding time for 2–5 s, tensile strength of the welded joints firstly increased and then decreased and the maximum tensile strength was obtained at 4 s, which was consistent with the observed results of microstructures and microhardness.

**Figure 14:**
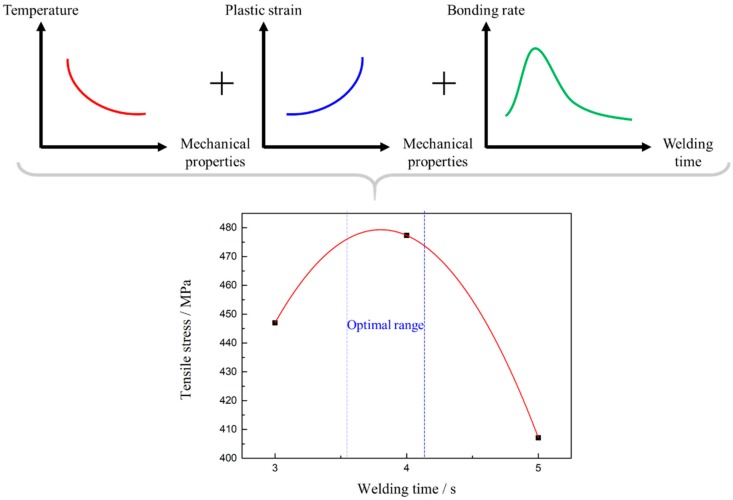
Effects of superposition of the three factors on quality of the joints.

Peak temperature near the frictional contact face during RFW of Mo increased with the extension of welding time, and the higher the temperature was, the higher the likelihood of the grain coarsening occurring in Mo, that is, the rise of peak temperature reduced properties of Mo joints. In the meanwhile, the maximum equivalent plastic strains near the frictional contact face increased in welding time, while violent plastic strain was beneficial for grain refinement and improvement of mechanical properties. Finally, this resulted in that mechanical properties of the joints firstly increasing and then decreasing with the extension of welding time. Similar phenomena have been reported by other scholars [28]. Some studies show that bonding rate (ratio of accumulated length of sound bonding region at friction interface to total length of friction interface) firstly rises and then reduces with the increase of welding time. It is also considered that when welding time is too long, the plastic zone near the contact surface of rotary friction is larger and the volume of metal extruded due to formation of flashes is greater. Once the extruded metal is not fully replenished, bonding rate and mechanical properties of the joints may be reduced. By combining with the test results in this study and those reported in literature, the mechanical properties of the Mo-RFW joints were the results of joint effects of three factors, i.e., temperature field, plastic strain, and balance between with the extrusion and replenishment of metal. As shown in Figure 14, temperature was inversely proportional to properties, while plastic strain and bonding rate were directly proportional to properties.

It is also noted that joint welded for 4 s was fractured in a parallel section far away from the weld seam (Figure 13b), while its tensile strength was obviously lower than that of base metal. This phenomenon may be because the thermal conductivity of molybdenum is about 3.2 times that of steel, and the 80 MPa welding pressure was relatively high. The relevant mechanisms need further study in the future.

### 3.6. Influences of Welding Time on Micro-Morphologies of Tensile Fractures of the RFW-Mo Joints

**Figure 15:**
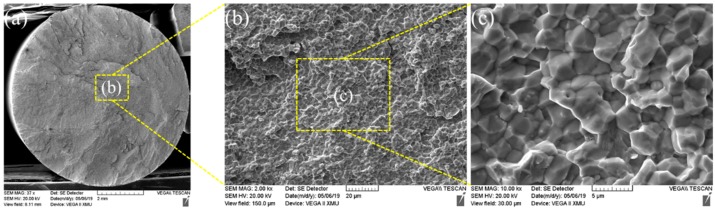
(**a**) Fracture morphology after welding for 3 s; (**b**) b region partial enlarged view of a; (**c**) c region partial enlarged view of b.

The fracture morphology of Mo-based metals is mainly quasi-cleavage, which has good toughness. Figure 15 demonstrates tensile fractures of the joint welded for 3 s, which showed SEM macro-morphology of the RFW-Mo specimens and high-magnification images of typical positions. Figure 13 shows the RFW-Mo joint for 3 s was fractured near the friction interfaces. The fractures observed with the SEM mainly showed intergranular brittle fractures. By combining with tensile curves in Figure 12, it can be found that there was no plastic stage during tensile test. During RFW process, when the temperature was over 900 °C, the recrystallization occurred to the molybdenum weld, and the strength of the grain boundary decreased from rolling state to recrystallization state. In addition, the intrinsic brittleness of molybdenum resulted in poor plasticity and intergranular fracture in SEM.

**Figure 16:**
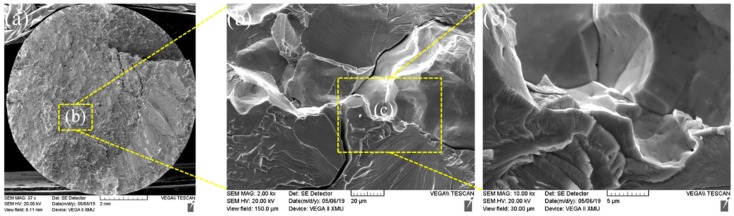
(**a**) Fracture morphology 1 after welding for 4 s; (**b**) b region partial enlarged view of a; (**c**) c region partial enlarged view of b.

**Figure 17:**
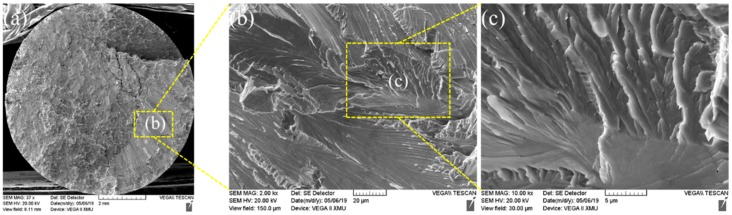
(**a**) Fracture morphology 2 after welding for 4 s; (**b**) b region partial enlarged view of a; (**c**) c region partial enlarged view of b.

Figure 16 and Figure 17 demonstrate morphologies of tensile fractures of the joint welded for 4 s. It can be observed from Figure 16 that large and flat cleavage morphologies were found in most areas of fracture. In addition, a small area of tensile fracture of the joint welded for 4 s showed river-pattern morphologies, as illustrated in Figure 17. The energy consumed during cracking of such river-pattern morphologies was greater than that consumed by forming large and flat cleavage morphologies, which may be an important reason for higher tensile strength of the joint welded for 4 s.

**Figure 18:**
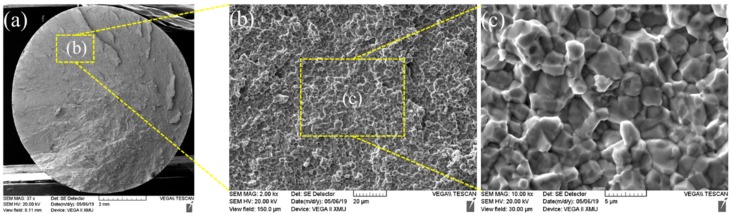
(**a**) Fracture morphology after welding for 5 s; (**b**) b region partial enlarged view of a; (**c**) c region partial enlarged view of b.

Figure 18 shows the morphologies of tensile fractures of the joint welded for 5 s. It can be observed from the figure that morphology of intergranular fracture was mainly shown in the fracture.

**Figure 19:**
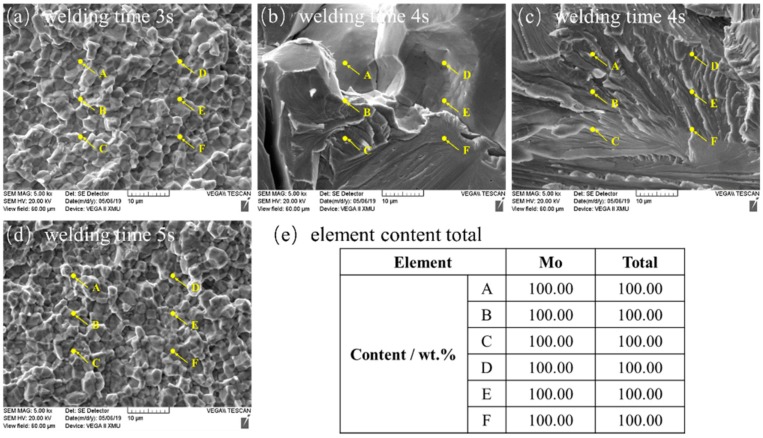
Results of energy spectrum analysis of the RFW-Mo joints: (**a**) welding time 3 s; (**b**) welding time 4 s-1; (**c**) welding time 4 s-2; (**d**) welding time 5 s; (**e**) element content total.

Finally, energy dispersive spectrometry (EDS) tests were conducted on chemical components on the fracture surface of the joint welded for 5 s and the results of EDS demonstrated that the components are 100 wt.% Mo elements in fracture (Figure 19). This indicates that although violent combustion occurred during RFW of Mo in atmospheric environment, the weld zone was not oxidized. This may benefit from the characteristic that plastic metal in the area near the interface during RFW was continuously extruded from the friction interface.

Based on the above results and discussions, it was deemed feasible to use RFW without upset forging to seal the last weld spots on the upper end plugs of fuel claddings made of Mo. In fact, the last weld spots on the upper end plugs of fuel claddings made of Mo have to be welded in the high-pressure inert atmosphere, which allows one to more easily obtain reliable welding quality.

## 4. Conclusions

This research studied the feasibility of using RFW without upset forging to seal the last weld spots on the upper end plugs of fuel claddings made of Mo. The results showed that this method has good feasibility when welding the spots on the upper end plugs of Mo fuel claddings. The main conclusions are made as follows: 

(1) Combustion-like phenomenon during RFW of Mo was very intense and the flashes were thrown out due to instability, appearing in a spiral shape. 

(2) Axial shortening of the RFW-Mo joints linearly rose with the increase of welding time. RFW of Mo had a small process window and slight changes in welding parameters can have great influences on joint morphology and axial shortening.

(3) The microstructures were refined in weld zones of the RFW-Mo joints near the radial center and average grain size in weld zones of the joints gradually increased along the radial direction from center to surface. 

(4) With the gradual increase of welding time from 2 s to 5 s during RFW of Mo, grain fineness number in weld zones of the joints near the radial center firstly rose and then reduced and the average grain size was minimum when welding time was 4 s. 

(5) During RFW of Mo, with the increase of welding time within 2–5 s, tensile strength firstly increased and then decreased. As welding time was 4 s, the maximum tensile strength was 477.34 MPa, which was 78.66% that of the base metal. 

(6) Under the test conditions in this study, the optimal welding parameters were welding pressure of 80 MPa, spindle speed of 2000 r/min, and welding time of 4 s. 

Finally, the authors have to admit that there were still some shortcomings in this study. For example, there was a through hole with a diameter of about 1 mm in the center of the upper end plugs of nuclear fuel rods, while the solid rods were used in this study. Furthermore, the flashes of the RFW joints of common metals were axially symmetrical, while those of the RFW-Mo joints had asymmetric, spiral shapes, which may lead to non-uniform structural properties of the joints along peripheral direction. However, this characteristic was not considered in this study in sampling and analysis. All of these need to be studied in the future.

## Figures and Tables

**Table 1:** Components of Mo1 (wt.%).

No.	Main Component	Impurity Content (≤)								
	Mo	Al	Ca	Fe	Mg	Ni	Si	C	N	O
Mo1	≥99.95	0.002	0.002	0.010	0.002	0.005	0.010	0.010	0.003	0.008

**Table 2:** Parameters of RFW of Mo.

NO.	Spindle Speed (r/min)	Welding Pressure (MPa)	Welding Time (s)
1	2000	80	2
2	2000	80	3
3	2000	80	4
4	2000	80	5
